# HIV and AIDS among adolescents who use drugs: opportunities for drug policy reform within the sustainable development agenda

**DOI:** 10.1002/jia2.25045

**Published:** 2018-02-27

**Authors:** Khalid Tinasti

**Affiliations:** ^1^ College of Arts and Humanities Swansea University Swansea United Kingdom; ^2^ Global Commission on Drug Policy Geneva Switzerland

**Keywords:** SDGs, drug policy, people who inject drugs, adolescents who use drugs, drug control conventions, HIV key population

## Abstract

**Introduction:**

The international community's commitment to halve by 2015 the HIV transmission among people who inject drugs has not only been largely missed, instead new HIV infections have increased by 30%. Moreover, drug injection remains one of the drivers of new HIV infections due to punitive responses and lack of harm reduction resourcing. In the midst of this situation, adolescents are a forgotten component of the global response to illegal drugs and their link with HIV infection. The Sustainable Development Goals (SDGs) present an opportunity to achieve the global objective of ending AIDS among adolescents who use drugs, by addressing the structural vulnerabilities they face be they economic, social, criminal, health‐related or environmental.

**Discussion:**

The implementation of the SDGs presents an opportunity to address the horizontal nature of drug policy and to efficiently address the drugs‐adolescents‐HIV risk nexus. Adolescent‐focused drug policies are linked to goals 1, 3, 4, 10, 16 and 17. Goals 3 and 16 are the most relevant; the targets of the latter link to the criminalization of drug use and punitive policy environments and their impact on adolescents' health and HIV transmission risks. Moreover, it presents an opportunity to include adolescent needs that are missing in the three drug control conventions (1961, 1971 and 1988), and link them with the provisions of the Convention on the Rights of the Child (1989). Finally, the six principles to deliver on sustainable development are also an opportunity to divert adolescents who use drugs away from criminalization and punitive environments in which their vulnerability to HIV is greater.

**Conclusions:**

Addressing HIV among adolescents who use drugs is an extremely complex policy issue depending on different sets of binding and non‐binding commitments, interventions and stakeholders. The complexity requires a horizontal response provided by the SDGs framework, starting with the collection of disaggregated data on this specific subgroup. Ending AIDS among adolescents who use drugs requires the implementation of national drugs and HIV plans based on the multi‐sectoral approach and the transformative nature of the SDGs, to provide a comprehensive response to the epidemic among this key affected subgroup.

## Introduction

Responding to paediatric HIV has been high on every national political agenda for the last decade, with encouraging results in terms of declining new infections among children by 70% between 2000 and 2015 1. Yet, adolescents aged 10 to 19 years old continue to pay a high toll to HIV in terms of new infections, AIDS‐related deaths and lack of access to antiretroviral therapy (ART) and other relevant services. This age group represented 260,000 [81,000 to 450,000] of the total 1,800,000 [1,600,000 to 210,000] new infections (all ages) in 2016, with new infections and AIDS‐related deaths expected to continue to increase 2. Among adolescents and young people, subgroups most at risk of acquiring HIV such as those who use drugs have not been adequately addressed yet, prompting an increase in HIV infections through injecting and non‐injecting drug use.

However, new HIV infections among adolescents are not all related to drug use, as illustrated in sub‐Saharan Africa, where new HIV infections are high among young women and adolescent girls. In South Africa alone, adolescent girls represented 24% of all new infections in 2012 3. International organizations, funders, civil society and national authorities are teaming to address the stigma, the policy barriers and the lack of gender‐ and age‐tailored interventions for this subgroup, while their investments in other subgroups, such as those who use drugs, remain marginal.

It is accurate, nevertheless, that the policy response to HIV among adolescents who use drugs (AWUD), at the international level, is extremely complex. The complexity of the response is based on multilayered interactions between a large set of conventions, political declarations and resolutions from the HIV and AIDS, drug control, and children's and human rights sectors. However, this complex response needs to be efficient, as it is necessary to address an alarming situation. While there is no disaggregated data on the prevalence of drug use or injection among adolescents, or on the prevalence of HIV among adolescents who use or inject drugs, people who inject drugs (PWID) of all age groups are the key population with new infections increasing by 30% between 2011 and 2015 4, while the 2011 political declaration on HIV and AIDS committed its signatory countries to halve the transmission among this population 5. Moreover, 86% of PWID who know their status did not have access to ART in 2013 6.

To achieve the *All In* 2020 targets 7 and end AIDS among AWUD by 2030, this commentary will focus exclusively on policy development by reviewing countries' obligations through legally binding conventions (Convention on the Rights of the Child (CRC), 1989; drug control conventions, 1961, 1971, 1988) as well as non‐binding commitments countries took through designated political declarations (on the world drug problem, 2009 as complemented by the UNGASS 2016 outcome document; on ending AIDS by 2030, 2016; and on the public health dimension of drugs at the WHA, 2017). It will then lay down the role of the Sustainable Development Goals (SDGs) as a general policy framework allowing a multi‐sectoral response to HIV among AWUD, before framing HIV and drug use within the SDGs agenda to illustrate the needed policy interventions to end AIDS among adolescents by 2030.

## Discussion

The situation of people who use drugs (PWUD), injecting and non‐injecting, adults or adolescents, varies from one country to another, depending on the nature of each national epidemic, the mode of consumption of drugs, but mostly on the policy framework. In 2014, there were 11.7 million (8.4 to 19.0 million) people who inject drugs (PWID) (aged 15 to 64) globally, of which 24% resided in Eastern Europe alone. This region is highlighted as it faces the largest number of new HIV infections through drug injection as a mode of transmission in the world, and concentrates countries that oppose in policy and in practice the evidence‐based harm reduction measures 8. For instance in the Russian Federation, the country with the highest injection burden in the world with 2 million PWID (1.8 to 2.2 million) 9, HIV prevalence among young people aged less than 25 in Moscow reached 12% in 2012 10, while it reached 37.4% – strongly linked to drug injection – among homeless AWUDs in Saint Petersburg 11. In comparison, in France, where harm reduction services were introduced in the 1990s, PWID represented 1% of all new HIV infections in 2014 12.

These national laws and policies to address drug use are interpreted from the drug control conventions (namely the Single Convention on Narcotic Drugs of 1961, the Convention on Psychotropic Substances of 1971 and the Convention against Illicit Traffic of 1988) 13. These conventions objectives, as stated in the non‐binding preamble, are: preserving the health and wellbeing of all; ensuring access to illegal drugs for medical and scientific needs; and fighting the “evil” of addiction. The articles of the conventions, which are legally binding for countries that ratified them, gave prominence to the latter and resulted in measures that harshly counter drug use through the “war on drugs” and the militarization of the response to drugs on a global scale. This approach to drug control among adolescents has resulted in more health and social harms than it provided solutions 14, as illustrated by the high burden of HIV and HCV among AWUD.

The 1988 Convention against illicit traffic is the only one that refers directly to underage minors, addressing the inclusion of minors in the illicit trade or the delivery of illicit drugs to them 15. These provisions are aligned, in the text, with the article 33 of the CRC 16. They differ in practice. Indeed, the CRC provision needs to be interpreted in terms of the whole convention: to be based on human rights and to have a positive impact on children and adolescents 17. The Committee of the Rights of the Child has further reminded that in most countries children do not have access to drug use‐related HIV prevention, and has called for harm reduction services to be available to children and AWUD, the decriminalization of drug use among this subgroup 18, as well as the need to take the best interest of adolescents as a primary consideration in all decisions that concern them 19.

And while the 2009 political declaration on countering the world drug problem does not address adolescents, the outcome document of UNGASS 2016 introduced a new chapter to address drugs and youth. Signatory countries approved to provide health services to dependent adolescent during custody or arrest, but also to address their age‐specific needs and other social determinants of their involvement in the drug trade, gang‐related violence and urban crime 20, elements that enhance their vulnerability to HIV as well. The UNGASS outcome document provides the first‐ever negotiated agreed language on youth and drugs at the international level, and aligns with the SDG agenda in promoting universal and equitable access to health services for youth. The 2016 political declaration on ending AIDS reminded that access to HIV services is hindered for adolescents in many settings. It called for policy environment to take into account the HIV vulnerability of this group, while providing comprehensive harm reduction services to PWID – the UNGASS 2016 outcome document has failed to mention harm reduction, some countries arguing that providing paraphernalia promotes drug use. It also set target dates and treatment coverage by region for adolescents living with HIV or in risk of infection 21.

During the Post‐2015 Development Agenda process, the HIV community advocated for the inclusion of the Fast‐Track Strategy as a target to end AIDS 22 – currently target 3.3 – while the drug policy community did not engage in the process. During the discussions of the UN General Assembly's Open Working Group (OWG), the response to drugs appeared on the 11th session under goal 3: “by 2030, eliminate narcotic drug and substance abuse” and goal 16: “significantly reduce the irresponsible trade in arms and conflict commodities, and reduce violence and other negative impacts associated with trade in illicit drugs.” 23 This prohibition‐based language was later reviewed by UN Member States who have failed to achieve their former commitments – in the 1998 and 2009 political declarations on the world drug problem – to eliminate or significantly reduce drug use globally. The final text under goal 3 is the current target 3.5: “strengthen prevention and treatment of substance abuse, including narcotic drug abuse […]” 24.

The SDGs being the framework within which all new policies need to develop 25, how to use them practically to address HIV among AWUD? Here are some ways forward using existing international agreements and mechanisms to achieve ending the epidemic among youth by 2030. Being integrated and indivisible, the SDGs cover AWUD and HIV through directly two targets – 3.3 and 3.5 – but their needs cut across all the goals, while they are influenced by far more targets.

Central to addressing HIV among AWUD is target 17.18, on the need to enhance data collection in developing countries, focusing on adolescent's health, mental health and substance use 26. For example, it remains difficult to assess the situation of growing methamphetamine use among adolescents in Myanmar, sparking a risk of an HIV epidemic through risky sexual behaviour 27, without clear and disaggregated data. The issue of data collection among this age group is a serious barrier to the HIV response in developed countries as well. Indeed, the current collection of data is based on arrests, seizures and access to treatment 28 mainly. For adolescents, the UN has called on countries to move beyond school surveys 29 to collect data. Therefore, the indicator 3.5.1 (Coverage of treatment interventions for substance use disorders to prevent substance abuse by 2030) will not support the aim of the target unless it is complemented by disaggregated data. The issue of metrics and data collection is central to the future drug policy architecture and the response to HIV among all PWID age groups, pushing countries to trigger the discussion on their global review 30.

The second most important barrier to ending AIDS among AWUD remains the equitable access to quality harm reduction, HIV and other healthcare services for this population. This is where the interaction between HIV programming, health resourcing and financing, youth protection and drug control culminate, and where the use of the SDG agenda becomes clearest on a programmatic and policy level. To achieve targets 3.3 (end AIDS) and 3.5 (prevent substance abuse) for adolescents, there is a need for an enabling policy environment to enhance access to effective harm reduction services. On the financial implications, Harm Reduction International calculated that shifting 10% of the USD 100 billion currently devoted to drug law enforcement would allow for the coverage of all harm reduction needs in the world by 2020, including for adolescents 31. This becomes more urgent since the HIV epidemic among PWID continues to increase while no additional country introduced needle and syringe programmes (NSP) since 2014. These evidence‐based services and their access are hindered by the criminalization of drug use 32, and are further refused to adolescents depending on the cultural and political contexts 33.

Moreover, the UNGASS 2016 outcome document and the 2009 drug control political declaration do not refer to harm reduction, countries not being able to get along on the use of this terminology during the negotiations for these documents. Thus, the implementation of the 2016 HIV political declaration in conjunction with drug control mechanisms at the national level is central to allow for the establishment of prevention and harm reduction services at the national level. Moreover, using the six principles to deliver on sustainable development 34 will allow for equitable access to quality services, as they call for people‐based policies. Such an approach, which is starting to take place in countries as diverse as Colombia, Thailand or Ghana results in policies that focus on the needs of PWUD rather than on eliminating the substance they use regardless of the negative impact on their health and human rights.

Finally, it is necessary to remind here that to prevent HIV infection and substance abuse among adolescents, better management of their mental health is necessary, since up to 20% experience mental health disorders starting the age of 14 35, and suicide rates are higher than among other age groups.

Another major interaction of SDGs to prevent HIV transmission among AWUD is the policy environment enabling the delivery of needed health services. This policy environment is decided through goal 16 and its targets' implementation. As currently illustrated in the Philippines, where the government envisages lowering the criminal liability age to 9 years old in an effort to enhance its “war on drugs” 36, the social determinants of the vulnerability of AWUD to organized crime and authorities' abuse are not addressed.

More practically, this means addressing the policy barriers for effective rights‐based responses to HIV among AWUD: the violence (16.1) and abuse (16.2) experienced by adolescents need to be effectively countered through fair access to justice and the rule of law (16.3), accountable institutions (16.6) and non‐discriminatory policies (16.b). In the case of the Philippines, the central government as well as provincial governments in charge of delivering health services need to work in collaboration with law enforcement, the judiciary, children protection services and social workers to provide an environment where AWUD are not afraid to seek services they need, that these services are available and of quality, that the criminal responsibility is lifted for use and possession of illegal drugs, and that the social and economic empowerment (10.2) and quality education (4.1) are pursued while protecting youth of legal liability for drug‐related small‐scale offences, in order to avoid putting them in a situation of risky sexual behaviour and prevent HIV infection and violence.

## Conclusions

Addressing HIV among adolescents who use drugs is an extremely complex policy issue, especially since it relies on different sets of binding and non‐binding commitments by countries, and on different sets of interventions provided by different stakeholders. It needs effective coordination as well as political will and commitment. It also requires better metrics to collect data on adolescents and drug use, adolescents with dependence, as well as data on access of AWUD to healthcare and social services in all regions of the world. Most importantly, it needs the inclusion of adolescents who use drugs themselves (along with the parent or legal guardian) in the design and implementation of drug policies, in accordance with the right of the child to be heard (article 12 of the CRC) and SDG target 16.7 on ensuring inclusive decision‐making.

The SDGs provide a framework allowing for a horizontal response, taking into account all needed policies, health and social interventions, and the legislative reforms. Ending AIDS among AWUD needs to be based on the implementation of the SDGs in national drugs and HIV plans, since the SDGs are a transformational agenda for all public policies, that no target is considered met until it is met for all economic and social groups – including by PLHIV, PWID and adolescents – and that all actions advance human rights. This is the only way forward in order to reach the AWUD furthest behind in the HIV response and not to leave anyone behind and, especially key populations and their subgroups.

## Competing interests

The author declares no competing interests.
